# Clinical Efficacy and Safety of Thai Herbal Formulation-6 in the Treatment of Symptomatic Osteoarthritis of the Knee: A Randomized-Controlled Trial

**DOI:** 10.1155/2020/8817374

**Published:** 2020-12-09

**Authors:** Nut Koonrungsesomboon, Saowaros Nopnithipat, Supanimit Teekachunhatean, Natthakarn Chiranthanut, Chaichan Sangdee, Sunee Chansakaow, Pramote Tipduangta, Nutthiya Hanprasertpong

**Affiliations:** ^1^Department of Pharmacology, Faculty of Medicine, Chiang Mai University, Chiang Mai 50200, Thailand; ^2^Musculoskeletal Science and Translational Research Center, Chiang Mai University, Chiang Mai 50200, Thailand; ^3^Center of Thai Traditional and Complementary Medicine, Faculty of Medicine, Chiang Mai University, Chiang Mai 50200, Thailand; ^4^Department of Pharmaceutical Sciences and Medicinal Plant Innovation Center, Faculty of Pharmacy, Chiang Mai University, Chiang Mai 50200, Thailand

## Abstract

**Background:**

Osteoarthritis of the knee is the most common form of arthritis. Identifying effective and safe herbal formulations that are locally available is viewed as a priority for sustainable development in a region. This study aimed to evaluate the efficacy and safety of Thai herbal formulation-6 (THF-6) in comparison with oral diclofenac in patients with moderate-to-severe osteoarthritis of the knee.

**Methods:**

This randomized, double-blind, active-controlled, noninferiority trial randomly assigned patients with osteoarthritis of the knee to receive either THF-6 or diclofenac for four weeks. The primary outcome measure was the change from baseline in knee pain as measured by a 100 mm visual analog scale (VAS). Secondary outcome measures included knee stiffness, a stair climb test, the Knee Injury and Osteoarthritis Outcome Score, and safety parameters. Outcomes were assessed on a biweekly basis. Modified intention-to-treat (MITT) and perprotocol (PP) analyses were applied.

**Results:**

A total of 200 patients were enrolled of whom 175 (87.5%) were included in the MITT analysis and 153 (76.5%) in the PP analysis. The mean change in VAS pain did not differ between the two groups, and the upper limit of the two-sided 95% confidence interval (CI) for comparison between the two groups was within the prespecified margin of 10 mm for noninferiority (MITT analysis: mean difference = 0.86, 95% CI = -4.39 to 6.10, *p*=0.748; PP analysis: mean difference = 1.98, 95% CI = -3.61 to 7.56, *p*=0.486). Significant improvement was observed in all the efficacy parameters in both groups. Dyspepsia was the most common adverse event: 23 patients in the THF-6 group and 28 in the diclofenac group (*p*=0.417).

**Conclusions:**

THF-6 offers an alternative to oral diclofenac for the short-term treatment of osteoarthritis of the knee. It was shown to be noninferior to oral diclofenac in relieving knee pain. This trial is registered with ChiCTR-IPR-15007213.

## 1. Introduction

Osteoarthritis is the most common form of arthritis, commonly affecting knee joints and causing pain, functional disability, and reduced quality of life [1, 2]. Osteoarthritis of the knee is one of the leading causes of global disability among older adults [3]. The burden of disease on the healthcare system, in addition to its impacts on individual patients, has been increasing worldwide over the past three decades [4, 5]. Nearly half of the population may develop symptomatic osteoarthritis of the knee by age 85, and the lifetime risk is doubled among obese individuals [6]. With increasing life expectancy, osteoarthritis of the knee is anticipated to cause even more economic challenges in the future that every country and international community needs to grapple with [7].

Nonsteroidal anti-inflammatory drugs (NSAIDs) are one of the most widely used medications for the management of knee pain in patients with osteoarthritis of the knee [8]. The efficacy of oral NSAIDs, diclofenac in particular, for symptomatic relief of knee osteoarthritis has been well established in the literature [9]. Thanks to their good analgesic and antiphlogistic effects, NSAIDs are recommended by several international and national guidelines as the initial oral medication of choice in the treatment of symptomatic osteoarthritis of the knee with moderate-to-severe pain intensity [10, 11]. Several NSAIDs, including oral diclofenac, are available as over-the-counter medications in many countries, so the use of this drug is particularly widespread [12, 13].

Given the large number of people affected with osteoarthritis of the knee and societal trends in population aging and obesity [14], identifying effective and safe therapeutic options that are available in a region is viewed as a priority for sustainable development in a country [15]. In this regard, herbal medicine could be a promising approach to be employed in developing such therapeutic options [16]. Several herbal extracts and formulations have been discovered and proven that they can bring about therapeutic benefits in terms of pain and mobility in patients with osteoarthritis of the knee [17–19]. In Thailand, the Thai herbal formulation-6 (THF-6) comprising six herbal materials, all of which are locally available in Thailand, has traditionally been used to augment longevity as well as for the treatment of muscle and joint pain [20–22]. Although anecdotal evidence exists supporting the efficacy and tolerability of THF-6 in the treatment of osteoarthritis of the knee [22], scientific proof of its benefits is required to verify its therapeutic potential.

The present study was designed to clinically assess the efficacy and safety of THF-6 in comparison with the standard drug, oral diclofenac, for the treatment of moderate-to-severe osteoarthritis of the knee.

## 2. Methods

### 2.1. Trial Design and Setting

This prospective, randomized, double-blind, double-dummy, and active-controlled trial was conducted at the Faculty of Medicine, Chiang Mai University, Thailand. The trial followed the OARSI Clinical Trials recommendations for the design, conduct, and reporting of clinical trials for osteoarthritis of the knee [23]. It was prospectively registered with the Chinese Clinical Trials Registry (ChiCTR-IPR-15007213). The clinical trial protocol and related documents were approved by the Research Ethics Committee of the Faculty of Medicine, Chiang Mai University (EC273/2015). The full-trial protocol (in Thai) is available from the corresponding author upon reasonable request. Written informed consent was obtained from all patients prior to their participation in the trial.

### 2.2. Trial Participants

Eligible patients were 45 years of age or older with osteoarthritis in one or both knees for more than three months. The disease was diagnosed according to the American College of Rheumatology criteria [24] with radiographic confirmation (Kellgren–Lawrence grade 2 or higher). Patients were eligible for inclusion if they had osteoarthritic knee pain of at least moderate intensity (defined as a pain score of ≥35 mm at baseline on a 100 mm visual analog scale (VAS)) [25]. Patients were excluded if they also had any other underlying arthritis (e.g., rheumatoid arthritis or gouty arthritis), signs or symptoms of active inflammation at the knee, a condition requiring knee surgery in the next few months, or a recent knee injury; had used intra-articular corticosteroid injections in the previous six weeks; had used symptomatic slow-acting drugs for osteoarthritis (e.g., glucosamine sulfate or chondroitin sulfate) within the previous four months or had discontinued those drugs for less than six months; had a history of an allergic reaction to oral NSAIDs or herbal ingredients in THF-6; or had a history of gastrointestinal ulcer, perforation, or hemorrhage. Other exclusion criteria were pregnancy or lactation and any clinically significant abnormalities of blood chemistry (including serum uric acid of >9 mg/dL) or other hematological parameters.

### 2.3. Sample Size Determination

With 69 patients per group, the trial was estimated to have 90% power at a two-sided alpha level of 0.05 based on a noninferiority margin of 10 [26, 27] and assuming a mean difference (MD) of 0 and a standard deviation (SD) of 20 [28]. Anticipating a 30% premature discontinuation or poor compliance, a total of 200 patients (100 per group) were planned to be enrolled in this trial.

### 2.4. Trial Interventions

The THF-6 (given at the dose of 1,500 mg/day) consisted of six herbs: fruit of *Streblus asper* Lour, stems of *Tinospora cordifolia* (Willd.) Miers, corms of *Cyperus rotundus*, bark of *Albizia procera* (Roxb.) Benth, bark of *Diospyros rhodocalyx* Kurz, and fruit of *Piper nigrum* Linn [21]. The components and preparation of THF-6 are summarized in Table S1. The THF-6 capsules (250 mg/capsule) were manufactured by the Department of Pharmaceutical Sciences and Medicinal Plant Innovation Center, Faculty of Pharmacy, Chiang Mai University, in compliance with the Thai Pharmacopoeia standards and requirements. High-performance liquid chromatography and thin-layer chromatography were used for quality control of THF-6.

Diclofenac (Voltaren^®^) (25 mg/tablet) was purchased from Olic Thailand limited and used as an active control in this trial. Placebo tablets of THF-6 and diclofenac were manufactured by the Department of Pharmaceutical Sciences and Medicinal Plant Innovation Center, Faculty of Pharmacy, Chiang Mai University.

### 2.5. Trial Procedures and Outcome Assessments

**Figure 1:**
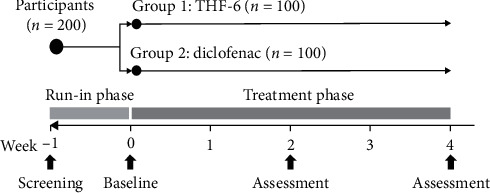
Study design.

The trial consisted of a one-week run-in phase followed by a four-week treatment phase (Figure 1). During the one-week run-in period, eligible patients were instructed to discontinue all pain relief medications (including NSAIDs and other analgesics). At the beginning of the treatment phase, eligible patients with at least moderate pain intensity were randomly assigned in a 1 : 1 ratio to receive either THF-6 (1,500 mg/day) or diclofenac (75 mg/day) (Figure 1). The treatment group assignment was based on a randomization list prepared in advance by independent research staff with the use of a computer-based random number generator. Sequentially numbered, opaque envelopes containing the list were employed to safeguard allocation concealment. The envelopes were opened only after each patient had met the eligibility criteria at the end of the run-in phase. Both patients and outcome assessors were blinded to the treatment allocation.

In the THF-6 group, patients took two capsules of THF-6 (250 mg/capsule) and one placebo diclofenac tablet three times a day after meals. In the diclofenac group, patients took two placebo THF-6 capsules and one diclofenac tablet (25 mg/tablet) three times a day after meals. Enrolled patients were instructed to avoid other analgesics, anti-inflammatory drugs (including other NSAIDs), and other treatment modalities (e.g., acupuncture) while participating in the trial. Omeprazole tablets (20 mg/tablet) were prepared as rescue therapy in case of adverse gastrointestinal consequences following the administration of the study drugs. Patients were prematurely withdrawn from the trial if they developed intolerable knee pain necessitating other medications or treatment modalities, used other analgesics or anti-inflammatory drugs, had severe adverse drug reactions or allergic reactions to the study drugs, or were lost to follow-up.

This trial assessed efficacy outcomes for pain, function, and global assessment according to the recommendations of a core domain set for outcome measurement in clinical trials of knee osteoarthritis [29, 30]. Outcome assessment was performed on a biweekly basis, that is, at the end of the one-week run-in phase (baseline) and at the end of Week 2 and Week 4 (Figure 1).

The primary efficacy metric was the change from baseline in knee pain as measured by a horizontal 100 mm VAS (rated on a scale of 0 to 100, with higher scores indicating worse knee pain) [31]. The secondary efficacy outcomes included VAS stiffness (rated on a scale of 0 to 100, with higher scores indicating more severe stiffness), a 10-step stair climb test (SCT) (time in seconds to ascend and descend a flight of 10 steps) [32], the Knee Injury and Osteoarthritis Outcome Score (KOOS) (which included 42 items across five domains; each item was rated on a 5-point Likert scale and transformed to a scale of 0 to 100, with higher scores indicating fewer knee problems) [33, 34], and the patient's and the physician's opinions of overall improvement on a 100 mm VAS (rated on a scale of 0 to 100, with higher scores indicating better improvement). In patients with bilateral osteoarthritis of the knee, efficacy outcomes were assessed only for the knee with worse symptoms at baseline.

Adverse events observed by the investigators or reported by the patients following a nondirective question were recorded. Drug compliance was assessed by counting returned unused medications at each visit. Any patient taking less than 70% of the allocated dose of study drugs was regarded as noncompliant.

### 2.6. Statistical Analysis

Analyses of efficacy outcomes were conducted using the perprotocol (PP) and the modified intention-to-treat (MITT) approaches, with the last observation carried forward method. For the safety evaluation, all patients who had received at least one dose of study drugs were analyzed. Statistical analysis was performed using SPSS version 22.0. A *p* value of <0.05 was considered to indicate statistical significance.

Continuous variables are presented as mean ± SD. Within-group comparisons were conducted to determine any differences in the mean values of each variable between baseline and the two consecutive follow-up visits; a repeated measures ANOVA, with the least significant difference (LSD) test, was applied. For between-group comparisons, mean changes from baseline were compared using Student's *t*-test. Patients were classified as having had a response if their VAS pain scores decreased by 50% or more from baseline [35]. Dichotomous variables are reported as frequencies; the chi-squared test or Fisher's exact test, as appropriate, was used to compare the distribution of dichotomous variables between the two groups.

For assessment of noninferiority, a comparison between the THF-6 group and the diclofenac group on VAS pain was conducted, with a prespecified noninferiority margin of 10 mm [26, 27]. Noninferiority was declared if the upper limit of the two-sided 95% confidence interval (CI) for the MD of VAS pain did not exceed a margin of 10 mm.

## 3. Results

**Figure 2:**
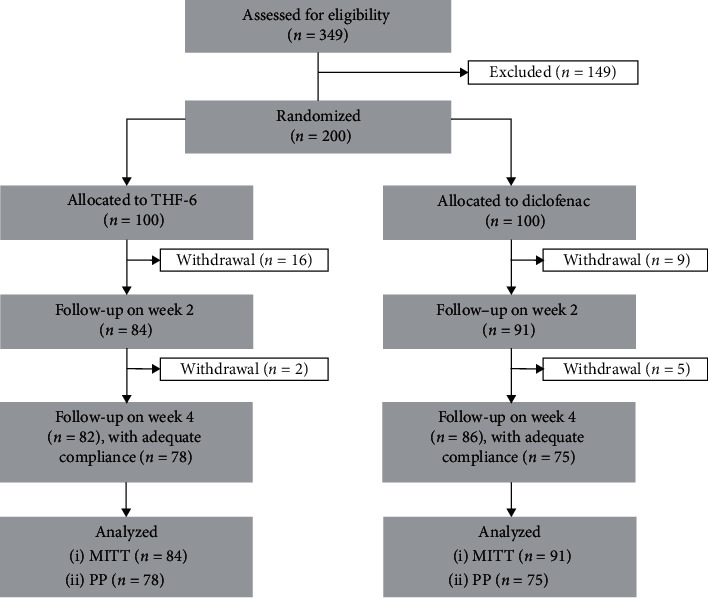
Flow diagram of the progress through all phases of this two-arm, randomized-controlled study (enrollment, intervention allocation, follow-up, and data analysis).

From February 2016 to July 2016, a total of 349 patients were screened for eligibility and 200 underwent randomization, 100 of whom were allocated to each arm (Figure 2). Predominant reasons for screening failure were that the patient did not meet the inclusion criteria (115 patients, 77.2%) and that the patient had abnormal laboratory results (31 patients, 20.8%). The mean age of the enrolled patients was 61.1 ± 6.1 years; 85.5% were women and 86% had osteoarthritis in both knees. Baseline demographic and clinical characteristics of the patients were comparable between the two groups (Table 1). Of the 200 patients randomized, 175 (87.5%) were included in the MITT analysis and 153 (76.5%) in the PP analysis (Figure 2).

**Figure 3:**
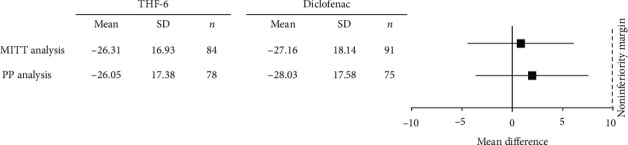
Noninferiority analysis of VAS pain.

With regard to the primary endpoint, the mean change in VAS pain was not statistically significantly different between the two groups, and THF-6 (1,500 mg/day) was found to be noninferior to diclofenac (75 mg/day) in both the MITT and PP analyses. The upper limit of the two-sided 95% CI for the comparison between the THF-6 group and the diclofenac group was within the prespecified margin of 10 mm for noninferiority (Figure 3).

None of the mean changes in any of the efficacy outcome measures differed statistically significantly between the two groups (Table 2). Significant improvements in all efficacy parameters among patients receiving THF-6 as well as those receiving oral diclofenac were observed by the end of Week 2, and significant improvements continued to be seen at the end of Week 4 (Figures S1 and S2). There were 42 responders (50%) at the end of the four-week treatment with THF-6 compared to 40 (44%) with oral diclofenac (MITT analysis: 42/84 vs. 40/91, *p*=0.423; PP analysis: 39/78 vs. 36/75, *p*=0.805). Overall improvement self-assessed by the patients was similar in both groups: no statistically significant difference between the two groups was observed at the end of the treatment phase (MITT analysis: 63.57 ± 22.46 vs. 64.20 ± 22.92, *p*=0.859; PP analysis: 63.65 ± 21.86 vs. 63.00 ± 23.71, *p*=0.859). Upon completion of this trial, the physician's assessment of overall improvement did not differ between the two groups (Figure S3).

Dyspepsia was the most common adverse event reported in both groups: 23 patients (23%) in the THF-6 group and 28 (28%) in the diclofenac group (relative risk = 0.821, 95% CI = 0.510 to 1.323, *p*=0.417). Overall, the proportion of patients reporting any adverse events in the safety population was fairly similar between the two groups (Table 3). All the adverse events reported were mild to moderate in intensity. Adverse events led to the premature discontinuation of 14 patients in the THF-6 group and 12 patients in the diclofenac group (*χ*^2^ (1, *n* = 200) = 0.177, *p*=0.674). Gastrointestinal intolerance was the major reason for discontinuation of therapy (9 in the THF-6 group vs. 6 in the diclofenac group, *χ*^2^ (1, *n* = 200) = 0.649, *p*=0.421). During the trial, there was only one serious adverse event, hospitalization due to pesticide exposure, which was judged as definitely unrelated to the study drug.

## 4. Discussion

In this randomized-controlled trial, THF-6 offered the potential to achieve analgesic efficacy with acceptable safety profiles in patients with moderate-to-severe osteoarthritis of the knee. In both the MITT and PP analyses, the upper limit of 95% CIs of MD in VAS pain was within the predefined noninferiority margin of 10 mm. This allowed us to conclude that THF-6 was noninferior to oral diclofenac (75 mg/day) in terms of knee pain relief. Oral administration of THF-6 also resulted in significant improvement, comparable to oral diclofenac, across several outcome measures. As THF-6 and oral diclofenac displayed comparable efficacy and safety, it seems appropriate to consider THF-6 an alternative for the treatment of moderate-to-severe osteoarthritis of the knee.

The present clinical trial was designed to evaluate the efficacy and safety of THF-6 versus oral diclofenac for the short-term treatment of symptomatic osteoarthritis of the knee. In clinical practice, pain relief medications, including oral NSAIDs, are typically prescribed on an as-needed basis for a short duration due to safety concerns [36]. Most randomized-controlled trials evaluating the effects of pharmacological interventions, especially oral NSAIDs, often last only a few weeks with only a small number of trials going beyond four weeks of treatment [26]. Therefore, the present trial design, with a treatment duration of four weeks, can be considered appropriate for determining the effects of THF-6 in symptomatic osteoarthritis of the knee.

With respect to osteoarthritic knee pain of moderate-to-severe intensity, this trial demonstrated that THF-6 was comparable to oral diclofenac in relieving pain symptoms. An average reduction of around 26 mm on a 100 mm VAS pain scale with THF-6 treatment can be considered a clinically important improvement [37]. A 50% decrease in pain score, which represents a reasonable cut-off value for indicating a clinically meaningful pain reduction from the patient's perspective, was observed in approximately half of the patients in the THF-6 group [35]. These findings support the efficacy of THF-6 in osteoarthritic knee pain relief.

Even though the precise mechanism of THF-6's action has not yet been elucidated, the herbal constituents in the formulation have been shown individually to possess several pharmacological activities, perceivably contributing to the beneficial effects of THF-6 in the treatment of symptomatic osteoarthritis of the knee. Previous comprehensive literature reviews of *C. rotundus* have found that the plant extracts have a broad range of pharmacological activities, including anti-inflammatory, antinociceptive, antiarthritic, and antioxidant activities [38–40]. *P. nigrum* also exhibits various pharmacological activities, for example, anti-inflammatory, antinociceptive, antiarthritic, and antioxidant activities, in both *in vitro* and animal experiments [41–43]. *S. asper* contains a number of bioactive compounds which possess anti-inflammatory and antioxidant properties [44, 45]. The crude extracts and isolated compounds of *T. cordifolia* have a wide range of pharmacological effects, including anti-inflammatory, antioxidant, antiarthritic, and analgesic properties [46, 47] as does *A. procera* [48–50]. As a result, it is reasonable to assume that the favorable outcomes following THF-6 administration in this trial might be attributable to the combined and possibly synergistic pharmacological activities of the several herbal ingredients in the formulation. Further research is warranted to ascertain the mechanisms of THF-6's action as well as understanding the molecular basis of its effects in symptomatic relief of osteoarthritic knee pain.

In this trial, the overall safety profiles of both pharmacological interventions were more or less the same. Around one-fourth of the patients in each group experienced gastrointestinal adverse events following drug administration. Dyspepsia was the most common adverse event in both groups and its frequency did not differ between the groups. Nine patients in the THF-6 group discontinued treatment early due to gastrointestinal adverse consequences, as did six patients in the diclofenac group. These findings are not surprising given that similar gastrointestinal tolerability problems with oral diclofenac, particularly dyspepsia, have been regularly observed in other clinical trials assessing the safety and tolerability of oral NSAIDs [51, 52]. Based on its safety profile as described above, THF-6 should be used with caution, especially in patients at high risk of upper gastrointestinal complications.

There were limitations to this trial. First and foremost, there was no placebo comparison in the trial where a subjective outcome measure was used as a primary endpoint. In the context where standard therapy is widely used to treat a condition, a placebo comparison might not be possible due primarily to ethical reasons [53]. In such a context, noninferiority design could be applied to compare the intervention under investigation with another active treatment provided that the reference treatment's efficacy is well established [54], as is the case for oral diclofenac [9]. This is of particular relevance in the present trial where the target groups had baseline knee pain of moderate-to-severe intensity. To minimize the risk of bias in the assessment of subjective outcomes, this trial assessed knee symptoms using several different measures to help ensure a comprehensive evaluation and also included a 10-step SCT measure which is less subject to contextual effects [55]. It should also be acknowledged that the MITT approach may be prone to attrition bias associated with the exclusion of some patients from the analysis [56, 57]. Notwithstanding, it does not necessarily bias trial results [58]. In noninferiority trials, a PP analysis typically yields a more conservative estimate of treatment effects; therefore, the MITT approach is less likely to have a significant impact on estimated treatment benefits especially when both PP and MITT analyses consistently support noninferiority, as is the case of the present study [59]. Lastly, it is notable that the trial length of four weeks may not be long enough to determine long-term adverse outcomes of THF-6, including cardiovascular complications [60]. On the basis of the evidence currently available, this trial does not guarantee the safety of long-term use of THF-6.

## 5. Conclusions

THF-6 offers an alternative to oral diclofenac for the short-term treatment of moderate-to-severe osteoarthritis of the knee. Administration of THF-6 was shown to be noninferior to oral diclofenac in relieving knee pain. None of the outcome measures assessed in this trial favored either THF-6 or oral diclofenac with respect to symptomatic osteoarthritis of the knee.

## Figures and Tables

**Table 1:** Demographic and clinical characteristics of the enrolled patients.

	THF-6 (*n* = 100)	Diclofenac (*n* = 100)
Age (years)	62.0 ± 6.1	60.3 ± 6.1
Female sex (%)	87	84
BMI (kg/m^2^)	26.0 ± 4.7	25.7 ± 6.3^a^

Location of osteoarthritis (*n*)		
Right knee	9	7
Left knee	5	7
Both knees	86	86

Kellgren–Lawrence grade (*n*)		
Grade 2	62	68
Grade 3	68	73
Grade 4	56	45

Duration of osteoarthritis of the knee (years)	4.9 ± 4.7	4.7 ± 4.3
Underlying disease (*n*)		
Hypertension	43	50
Dyslipidemia	27	30
Diabetes mellitus	13	12
Miscellaneous	7	12
None	49	37

Baseline measures		
VAS pain (mm)	60.0 ± 14.8	61.9 ± 15.7
VAS stiffness (mm)	54.9 ± 20.2	56.9 ± 22.3
KOOS		
Pain	49.5 ± 14.3	51.4 ± 17.1
Other knee symptoms	55.2 ± 15.3	53.7 ± 18.3
Activities of daily living	51.3 ± 16.7	53.3 ± 18.3
Sport and recreation function	24.4 ± 19.4	26.8 ± 20.4
Knee-related quality of life	32.1 ± 15.8	32.0 ± 17.6
SCT (sec)	12.1 ± 5.9	11.5 ± 5.6

^a^ *n* = 99. KOOS, Knee Injury and Osteoarthritis Outcome Score; SCT, 10-step stair climb test; VAS, visual analog scale.

**Table 2:** Efficacy outcome assessments.

	THF-6	Diclofenac	Mean difference	(95% CI)	*p* value^a^
Mean change of VAS pain (mm)					
MITT analysis	−26.31 ± 16.93	−27.16 ± 18.14	0.86	(−4.39 to 6.10)	0.748
PP analysis	−26.05 ± 17.38	−28.03 ± 17.58	1.98	(−3.61 to 7.56)	0.486

Mean change of VAS stiffness (mm)					
MITT analysis	−22.52 ± 17.70	−23.26 ± 19.12	0.74	(−4.77 to 6.25)	0.791
PP analysis	−22.53 ± 17.82	−23.28 ± 18.10	0.75	(−4.98 to 6.49)	0.795

Mean change of SCT (sec)					
MITT analysis	−3.35 ± 3.55	−3.42 ± 4.33	0.07	(−1.12 to 1.26)	0.148
PP analysis	−3.44 ± 3.61	−3.27 ± 4.09	−0.17	(−1.40 to 1.06)	0.786

Mean change of KOOS pain					
MITT analysis	14.88 ± 14.98	15.77 ± 15.89	−0.89	(−5.50 to 3.73)	0.705
PP analysis	15.24 ± 15.29	15.77 ± 14.54	−0.53	(−5.30 to 4.24)	0.827

Mean change of KOOS other symptoms					
MITT analysis	12.29 ± 14.92	15.29 ± 16.90	−3.00	(−7.77 to 1.77)	0.216
PP analysis	12.49 ± 15.17	16.01 ± 16.22	−3.53	(−8.54 to 1.49)	0.167

Mean change of KOOS activities of daily living					
MITT analysis	13.25 ± 15.22	14.46 ± 17.78	−1.21	(−6.17 to 3.75)	0.640
PP analysis	13.62 ± 15.23	14.05 ± 16.76	−0.44	(−5.55 to 4.67)	0.866

Mean change of KOOS sport and recreation function					
MITT analysis	15.18 ± 17.28	18.57 ± 21.23	−3.39	(−9.20 to 2.41)	0.250
PP analysis	15.64 ± 17.48	19.27 ± 21.79	−3.63	(−9.91 to 2.66)	0.256

Mean change of KOOS knee-related quality of life					
MITT analysis	8.42 ± 16.23	13.71 ± 19.65	−5.30	(−10.70 to 0.10)	0.054
PP analysis	9.38 ± 16.30	13.95 ± 18.01	−4.56	(−10.05 to 0.92)	0.102

^a^Student's *t*-test. KOOS, Knee Injury and Osteoarthritis Outcome Score; SCT, 10-step stair climb test; VAS, visual analog scale.

**Table 3:** Safety outcome assessments.

Adverse events	THF-6 (*n* = 100)	Diclofenac (*n* = 100)	*p* value^a^
Gastrointestinal system			
Dyspepsia	23	28	0.417
Nausea and vomiting	3	4	1.000
Gastroesophageal reflux disease	2	2	1.000
Loss of appetite	4	1	0.369
Diarrhea	3	1	0.621
Constipation	5	5	1.000
Hematochezia	1	0	1.000

Other systems			
Peripheral edema	4	5	1.000
Peripheral neuropathy	2	1	1.000
Dizziness	1	2	1.000
Headache	1	0	1.000
Drowsiness	1	0	1.000
Insomnia	0	1	1.000
Dry mouth	0	1	1.000
Blurred vision	1	0	1.000
Low back pain	2	2	1.000
Myalgia	2	0	0.497
Cramp	1	1	1.000
Rashes	2	0	0.497
Itching	2	0	0.497
AST elevation (>2.5–3.0 times from baseline)	2	1	1.000
Creatinine rising (>1.5–2.0 times from baseline)	0	3	0.246

^a^Chi-square test or Fisher's exact test. AST: aspartate aminotransferase.

## Data Availability

All data used to support the findings of this study are available from the corresponding author upon reasonable request.

## Supplementary Materials

Supplementary Materials Table S1: components of Thai herbal recipe-6 (THF-6). Figure S1: VAS pain, VAS stiffness, and SCT at baseline, Week 2, and Week 4. Figure S2: KOOS at baseline, Week 2, and Week 4. Figure S3: Patient's and physician's opinion of overall improvement.
