# Supplementary material for: Clinical Efficacy and Safety of Thai Herbal Formulation-6 in the Treatment of Symptomatic Osteoarthritis of the Knee: A Randomized-Controlled Trial
Source: Evid Based Complement Alternat Med. 2020 Dec 9;2020:8817374. doi: 10.1155/2020/8817374 (PMC7749772; doi:10.1155/2020/8817374)
Supplement: Supplementary Materials — Table S1: components of Thai herbal recipe-6 (THF-6). Figure S1: VAS pain, VAS stiffness, and SCT at baseline, Week 2, and Week 4. Figure S2: KOOS at baseline, Week 2, and Week 4. Figure S3: Patient's and physician's opinion of overall improvement. [file 8817374.f1.docx]

**Table S1:** Components of Thai herbal recipe-6 (THF-6).

| **Common name** | **Scientific name** | **Family** | **Part used** | **Ratio** |
| --- | --- | --- | --- | --- |
| Black pepper | *Piper nigrum* L. | Piperaceae | Fruit | 1 |
| Heart-leaved moonseed or Guduchi | *Tinospora cordifolia* (Willd.) Miers | Menispermaceae | Stem | 1 |
| White siris | *Albizia procera* (Roxb.) Benth. | Fabaceae | Bark | 1 |
| Takona | *Diospyros rhodocalyx* Kurz | Ebenaceae | Bark | 1 |
| Siamese rough bush or  Khoi | *Streblus asper* Lour. | Moraceae | Seed | 1 |
| Nut grass | *Cyperus rotundus* L. | Cyperaceae | Rhizome | 1 |
| Honey | As binder | | | |


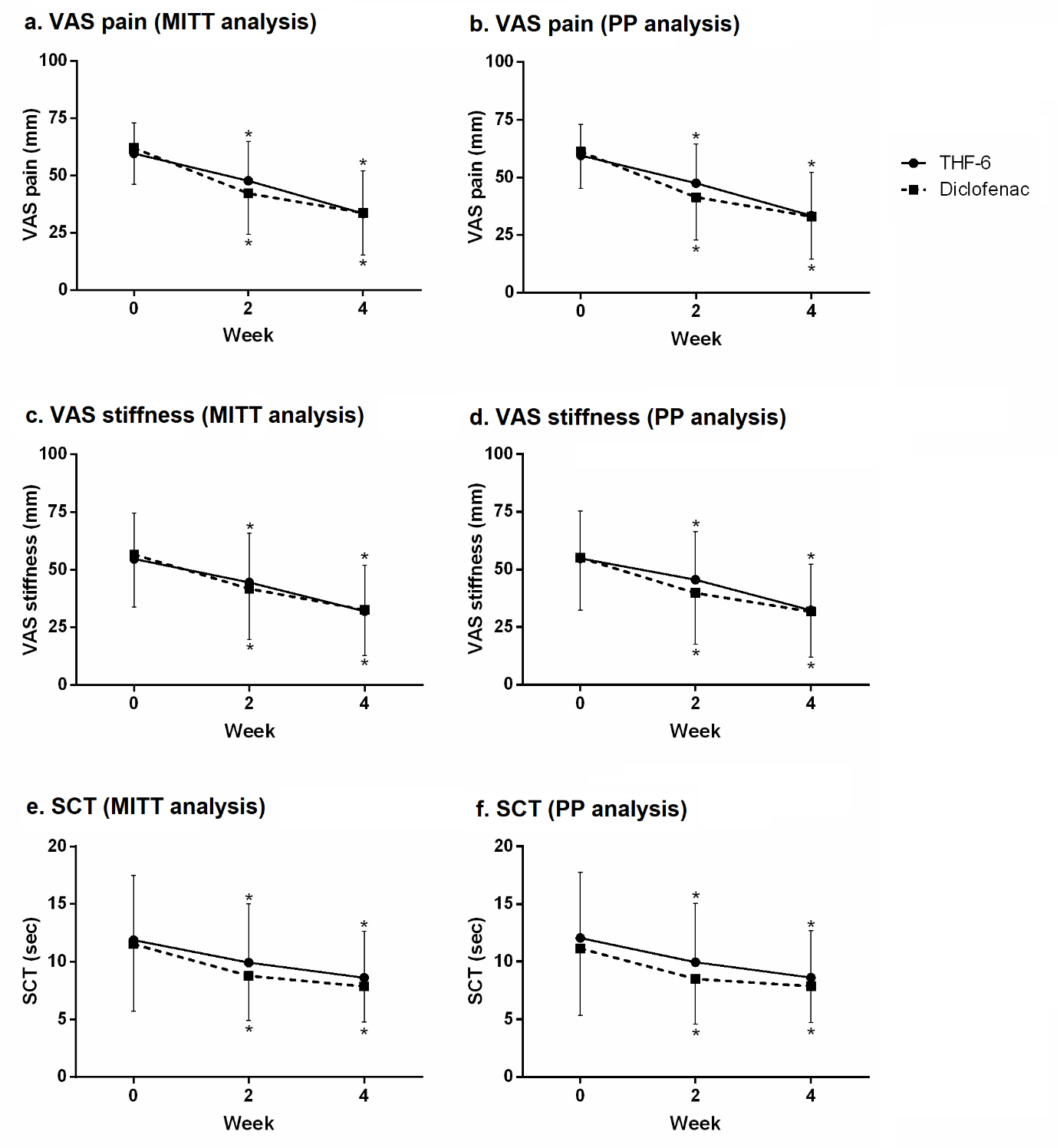


**Figure S1:** VAS pain, VAS stiffness, and SCT at baseline, week 2, and week 4. **p* <0.05 versus baseline value (one-way repeated measures ANOVA, followed by LSD test).


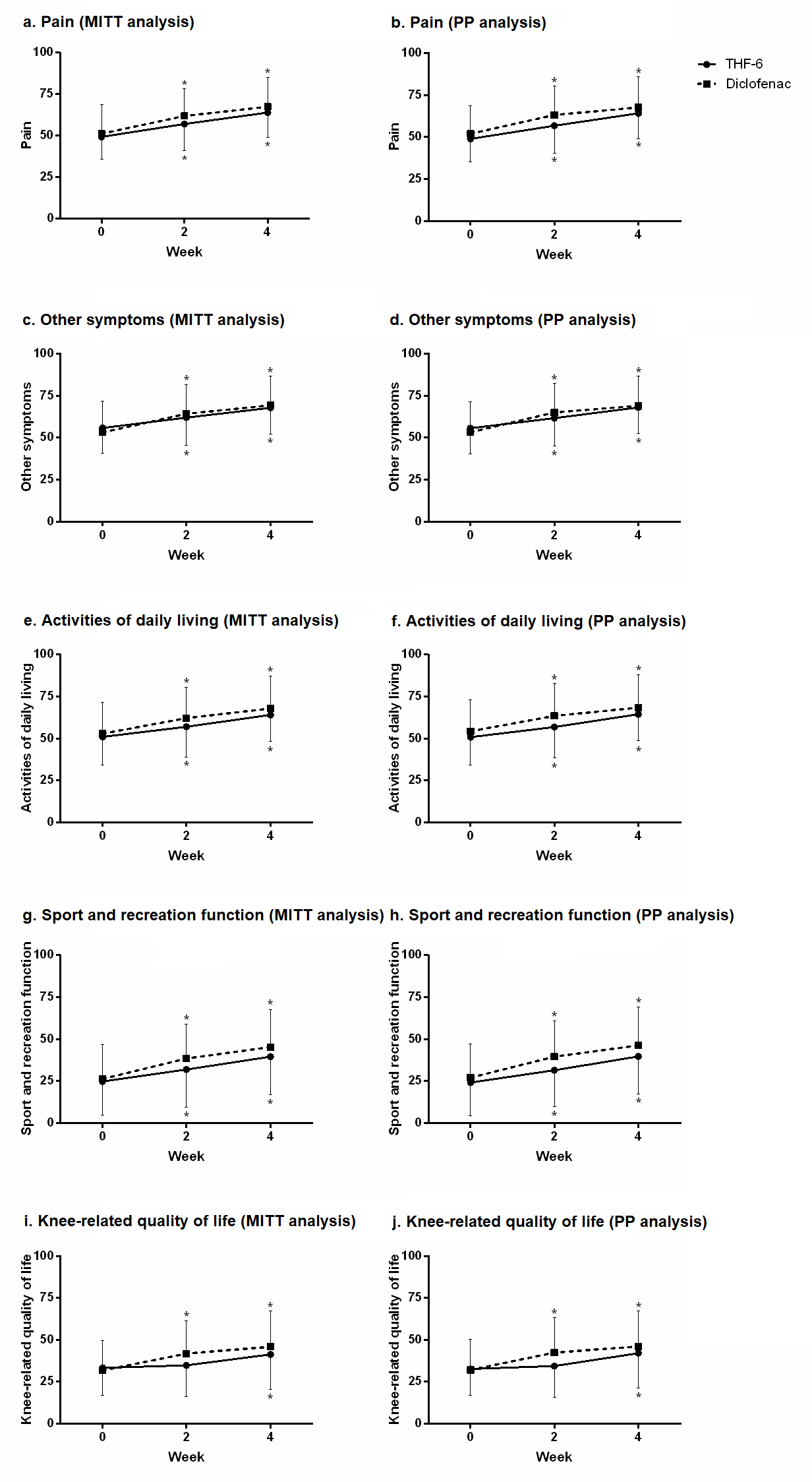


**Figure S2:** KOOS at baseline, week 2, and week 4. **p* <0.05 versus baseline value (one-way repeated measures ANOVA, followed by LSD test).


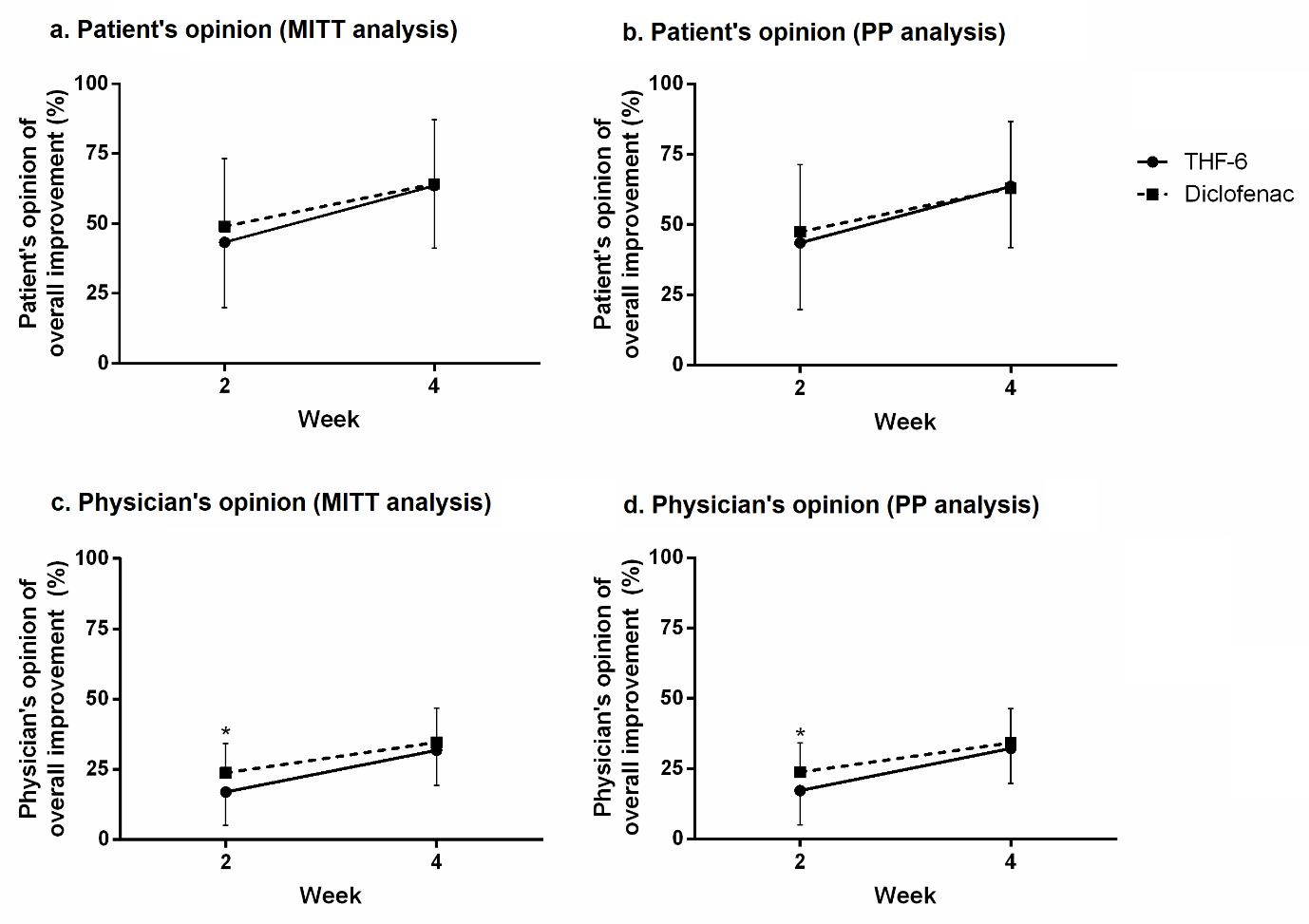


**Figure S3:** Patient’s and physician’s opinion of overall improvement. ^*^*p* <0.05 versus Diclofenac (Student’s t-test)
